# The activation fragment of PAR2 is elevated in serum from patients with rheumatoid arthritis and reduced in response to anti-IL6R treatment

**DOI:** 10.1038/s41598-021-03346-0

**Published:** 2021-12-20

**Authors:** Stefania Kalogera, Yi He, Anne-Christine Bay-Jensen, Thorbjørn Gantzel, Shu Sun, Tina Manon-Jensen, Morten Asser Karsdal, Christian S. Thudium

**Affiliations:** 1 Nordic Bioscience, Immunoscience, Biomarkers and Research, Herlev, Denmark; 2 Department of Drug Design and Pharmacology, Copenhagen University, Copenhagen, Denmark; 3 Orthopaedic Surgery Unit, Gentofte University Hospital, Gentofte, Denmark

**Keywords:** Biochemistry, Biomarkers, Diseases, Medical research, Rheumatology

## Abstract

Osteoarthritis (OA) and rheumatoid arthritis (RA) are serious and painful diseases. Protease-activated receptor 2 (PAR2) is involved in the pathology of both OA and RA including roles in synovial hyperplasia, cartilage destruction, osteophyogenesis and pain. PAR2 is activated via cleavage of its N-terminus by serine proteases. In this study a competitive ELISA assay was developed targeting the 36-amino acid peptide that is cleaved and released after PAR2 activation (PRO-PAR2). Technical assay parameters including antibody specificity, intra- and inter-assay variation (CV%), linearity, accuracy, analyte stability and interference were evaluated. PRO-PAR2 release was confirmed after in vitro cleavage of PAR2 recombinant protein and treatment of human synovial explants with matriptase. Serum levels of 22 healthy individuals, 23 OA patients and 15 RA patients as well as a subset of RA patients treated with tocilizumab were evaluated. The PRO-PAR2 antibody was specific for the neo-epitope and intra-inter assay CV% were 6.4% and 5.8% respectively. In vitro cleavage and matriptase treated explants showed increased PRO-PAR2 levels compared to controls. In serum, PRO-PAR2 levels were increased in RA patients and decreased in RA patients treated with tocilizumab. In conclusion, PRO-PAR2 may be a potential biomarker for monitoring RA disease and pharmacodynamics of treatment.

## Introduction

The arthritides are a heterogeneous group of joint diseases with two of the most common being osteoarthritis (OA) and rheumatoid arthritis (RA). While different, both diseases are painful with a heavy impact on quality of life of patients.

OA is the most prevalent musculoskeletal disease globally^1^ and it is estimated that this chronic debilitating condition affects about 7% of the population worldwide. The pathogenesis of such a heterogenous disease is complex with different genetic, structural, mechanical, metabolic and inflammatory pathways involved in its progression^2^. OA affects the entire structure of the joint, with the most common hallmarks involved in OA pathology being cartilage degradation, osteophytes formation, subchondral bone remodeling and synovitis^3^. RA is the most common systemic inflammatory disease with a prevalence up to 1% worldwide with a peak at the age between 30 and 50 years old^4^*.* It is a multifactorial disease, like OA, but with a higher genetic pathological background. RA is mostly characterized by inflammatory pathways leading to proliferation of synoviocytes (hyperplasia) and subsequently to cartilage destruction and bone erosions. As an autoimmune disease, RA is, also, characterized by the presence of autoantibodies, mostly against rheumatoid factor and citrullinated peptide^5^.

While the treatments for OA and RA have been revolutionized over the last decades with the development of novel biologics, there are still large differences in the response to treatment among patients. Thus, there is a need for better and more specific markers that can aid in facilitating personalized treatment and increase treatment response^6^. An important tool for monitoring both OA and RA disease and increase treatment response could be through the application of precise biochemical markers. Although large efforts have been directed at the development of novel biomarkers in OA and RA^7^, there is still an unmet need for validated and qualified biomarkers released from different tissues of the joint, such as cartilage, bone, and synovium to describe joint structure pathology and inflammation^6^. Moreover, inflammatory markers like cytokines or neuropeptides could act as nociceptive sensitizers and be associated directly or indirectly with pain mechanisms^8^.

Protease-activated receptor 2 (PAR2) is part of the family of transmembrane, G-protein-coupled receptors. These receptors are activated by serine protease-mediated cleavage of their N terminus where the unveiling N-terminus acts as a “tethered ligand” that can bind the extracellular second loop and initiate signaling. PAR2 is expressed in a range of different tissues including kidney, pancreas, stomach, intestine, skin, brain. Here it is found in epithelial and endothelial cells, myocytes, fibroblasts, immune cells, neurons and glial cells as well as in immune cells including dendritic cells, lymphocytes, mast cells, neutrophils and eosinophils^9^. It is activated by a number of serine proteases such as trypsin^10^, mast cell tryptase^11^, neutrophil proteinase 3^12^ ,and matriptase^13^, many of which are generated and released during tissue injury and/or inflammation.

It is well described in the literature that inflammation is the primary feature in RA, whereas it contributes as well to the pathogenesis of OA. In both diseases different cytokines (TNF-α, IL-1, IL-6)^14–16^ stimulate the release of proteases, like serine proteases, matrix metalloproteinases (MMP-s), aggrecanases^17^. In OA inflammation is mostly localized in synovial membrane, while in RA affects more peripheral joints and leads to systemic inflammatory levels. PAR2 has been shown to contribute to inflammation in both OA and RA. Upon activation of PAR2, signaling through ERK1/2 and NF-κB pathways leads to the production of pro-inflammatory factors and cytokines including IL-1, TNF-α, IL-6, IL-8, as well as MMPs such as MMP-1 and MMP-13. So, in this way PAR2 is mostly contributing to disease progression through regulating production and release of various cytokines^18^.

It has been shown so far that PAR2 is overexpressed in OA chondrocytes compared to normal as well as in RA synovium^19^. Furthermore, correlation of PAR2 with synovitis was demonstrated by scoring of synovial thickness and monocytes infiltration^20^. This association, together with the observation that inhibiting PAR2 activation reduced TNF generation in a dose-dependently way, supports a pro-inflammatory role for PAR2 in synovial pathology. Other studies have investigated the effect of PAR2 antagonists in a pre-clinical aspect and showed that PAR2 blockage reduced disease progression and improved synovitis in adjuvant-induced monoarthritic mice models^21^. In addition to that, Yan et al. found that down-regulation of PAR2 could ameliorate OA through suppressing the release of inflammatory factors and proteases and promoting chondrocytes proliferation via regulation of MAPK/NF-κB signaling^22^. Furthermore, apart from PAR2 contribution to synovitis and cartilage destruction, a mice study from 2015 has been clearly shown that PAR2 contributes to osteophyte formation via its presence in proliferative and hypertrophic chondrocytes^23^. Finally, as an inflammatory mediator, there is strong evidence that PAR2 is involved in nociception and hyperalgesia^24^. Indeed, mice pain studies with pain behavioral tests have shown different patterns of dynamic weight-bearing in PAR2^−/−^ and wild type mice^23^.

In light of this, inhibiting the activation of PAR2 has also been suggested as a potential target for treatment of both OA and RA^25^. In this study we hypothesized that levels of PAR2 pro-fragment (PRO-PAR2) in systemic circulation may reflect PAR2 activation. An ELISA assay targeting the neo-epitope of the 36-amino-acid peptide released after PAR2 is cleaved and activated was developed and validated in serum as a novel biomarker for arthritic diseases.

## Results

### Generation of monoclonal antibodies

Monoclonal antibodies were generated by mice immunization with the selection peptide QGTSRSSKGR. This is the 10 amino acid target sequence adjacent to the PAR2 N-terminal cleavage site. Cleavage at this site generates a neo-epitope, which is a region of the protein that is generated only after cleavage of the full length PAR2 protein. The target sequence QGTSRSSKGR was blasted for homology to other proteins using NPS@: Network Protein Sequence and was found to be unique to the entire human genome. Antibody clones, produced after fusion between mouse spleen cells and myeloma cells, were tested for their reactivity, affinity, and stability. Based on reactivity, the antibody clone NBH-8B11-2E5-1B5 was selected for the assay development.

### Antibody specificity

**Figure 1:**
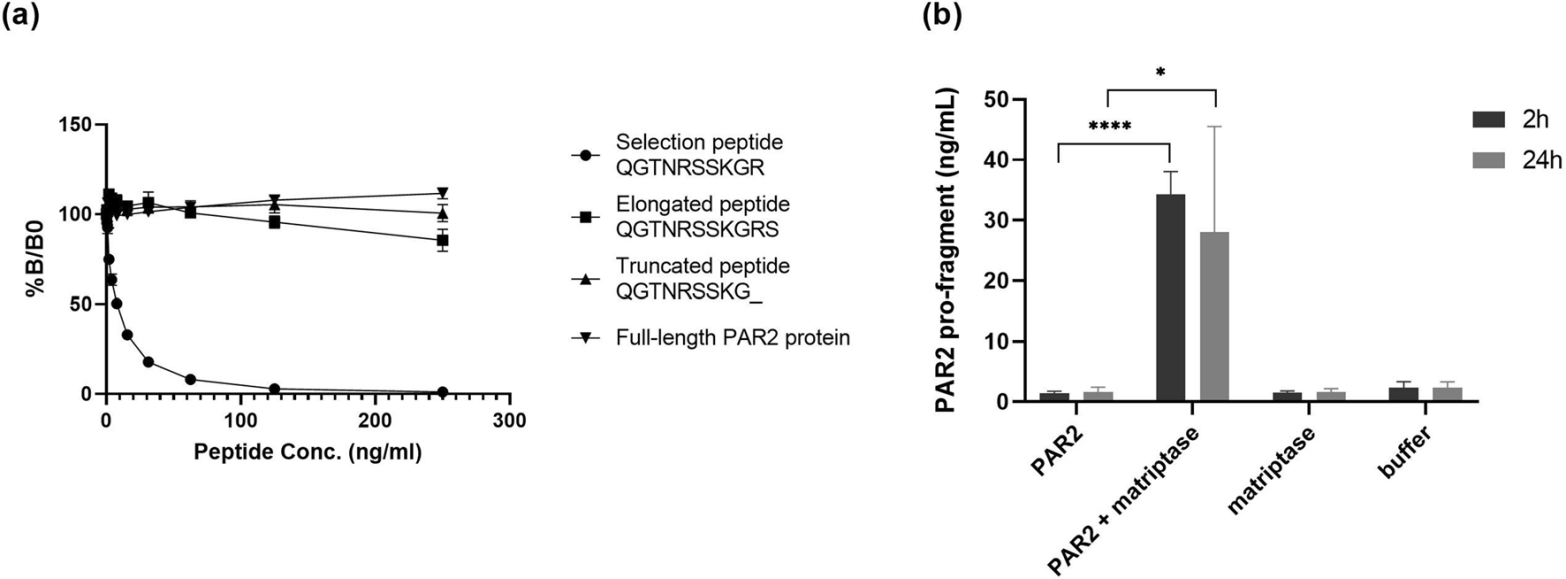
(**a**) Specificity of the monoclonal PAR2 antibody. Monoclonal antibody reactivity towards the selection peptide (QGTSRSSKGR), the elongated peptide (QGTSRSSKGRS), the truncated peptide (QGTSRSSKG) and the full-length PAR2 protein was tested. Two-fold dilutions of the peptides and the PAR2 protein were added starting from 250 ng/ml. The data suggest that the monoclonal antibody is specific only for the selection peptide, which is the target sequence. Data are shown as B/B0, where B represents the RLU at given concentrations and B0 the RLU at 0 ng/ml peptide and are presented as mean ± SD. RLU: relative luminescence units. (**b**) In vitro cleavage of recombinant full-length PAR2 protein with matriptase. Human full length PAR2 protein was incubated with matriptase for 2 and 24 h and PRO-PAR2 levels were quantified with the PAR2 immunoassay. Group comparisons were done using one-way ANOVA with Dunnett’s multiple comparisons. Data are presented as mean ± SD. Statistical significance is considered as *P < 0.05 and ****P < 0.0001.

Antibody specificity towards the neo-epitope was tested against the selection peptide, which is the 10 amino acid peptide sequence used to generate the monoclonal antibody, against synthetic peptides and against the full-length PAR2 protein. No reactivity was found towards neither the elongated and the truncated peptide nor towards the full-length PAR2 protein as the antibody only reacted with the selection peptide (Fig. 1a).

To characterize the generation of the PRO-PAR2 from full-length PAR2 and further evaluate the specificity of the antibody, the ability of matriptase to generate the PRO-PAR2 was assessed by enzymatic cleavage. Digestion with matriptase increased the levels of PRO-PAR2 after both 2 and 24 h, while no signal was observed in non-cleaved samples (Fig. 1b).

### Technical performance

Technical validation tests were carried out to evaluate the performance of the PRO-PAR2 assay. A summary of technical tests is provided in Table 1. The assay measurement range (LLMR–ULMR) was determined to be between 2.7 and 56.7 ng/ml. The calculated average inter- and intra-assay variations based on 10 independent runs were 6.4% and 5.8% recovery, respectively. Dilution recovery experiments were acceptable in the linear measurement range with an average recovery of 110.3% showing that the assay is accurate and robust. The analyte stability was acceptable for freeze/thaw cycles and prolonged storage of human serum samples in all tested conditions. Spiking of the full length PRO-PAR2 in serum resulted in a mean recovery of 108.8%. There was no interference with the assay from biotin, hemoglobin, and lipids in human serum.

**Table 1:** Summary of the technical validation of the PAR2 immunoassay.

Assay parameters	Results
Measurement range (LLMR–ULMR)	2.7–56.7 ng/mL
IC50	11.5 ng/mL
LLOD	1.5 ng/mL
Slope	1.15
Inter-assay variation	6.4% (4.1–8.6)
Intra-assay variation	5.8% (2.3–9.2)
Dilution recovery in serum and plasma	110.3% (107–115)
Spiking recovery (full-length peptide in serum)	108.8% (96–122)
Freeze and thaw cycles stability (4 cycles)	87.1% (84.8–88.4)
**Analyte stability**	
2 h, 4 °C/20 °C	98.6%/93.5%
4 h, 4 °C/20 °C	107.3%/102.7%
24 h, 4 °C/20 °C	113.4%/106.7%
48 h, 4 °C/20 °C	105.6%/120.3%
Interference hemoglobin, low/high	97.1%/92.4%
Interference lipid, low/high	115.2%/112.2%
Interference biotin, low/high	90.7%/79.3%

Percentages are reported as means (range). LLMR: lower limit of measurement range, ULMR: upper limit of measurement range, IC50: half maximum of inhibitory concentration, LLOD: lower limit of detection.

### Biological validation

#### PRO-PAR2 release from human synovial membrane explants

**Figure 2:**
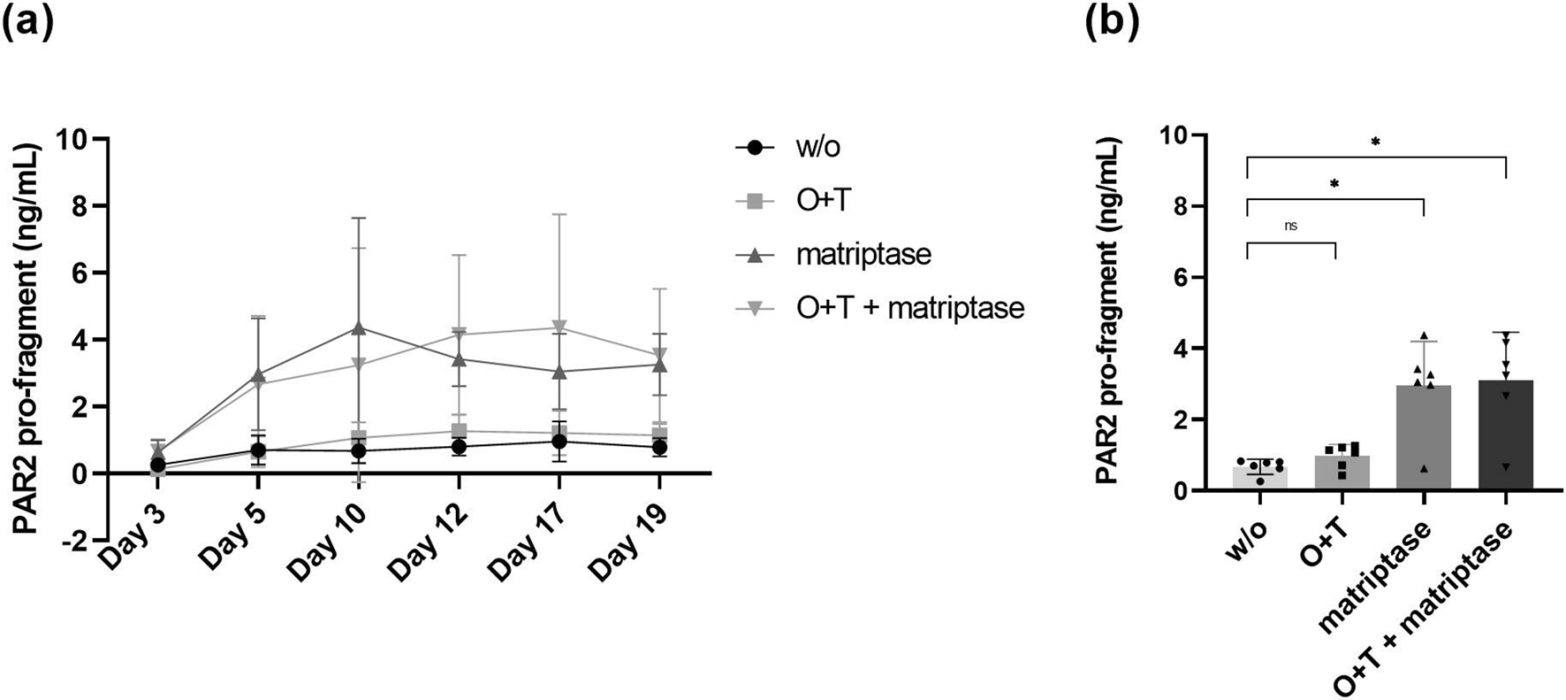
PRO-PAR2 release from human synovial explants (HEX). (**a**) PRO-PAR2 levels were measured in supernatant from the HEX model harvested on day 3, 5, 10, 12, 17 and 19. HEX cultures were either without treatment (w/o) or treated with matriptase and/or OSM + TNF-α (O + T). The release of the neo-epitope was quantified with the PAR2 immunoassay. (**b**) AUC analysis was performed to assess any statistically significant increase of PRO-PAR2 levels in matriptase-stimulated, (O + T)-stimulated as well as in matriptase + (O + T)-stimulated explants compared to the untreated explants. Group comparisons were done using Kruskal–Wallis test with Dunn’s post hoc for multiple comparisons. Data are presented as mean ± SD. Statistical significance is considered as *P < 0.05 and ns = *P* > 0.05.

To test whether the PRO-PAR2 can be released from the synovial membrane fibroblasts, PRO-PAR2 was quantified in the conditioned media of synovial membrane explants stimulated with matriptase and/or oncostatin M (OSM) and tumor necrosis factor α (TNF-α) (O + T). The pro-fragment was increased from day 5 in the conditioned media of matriptase-stimulated culture supernatants (Fig. 2a). When calculating the area under the curve (AUC) (Fig. 2b) there was a statistically significant increase in PRO-PAR2 in matriptase-treated explants with or without O + T compared to untreated (*p* = 0.019 and *p* = 0.030, respectively). No effect of stimulation with O + T on the levels of PRO-PAR2 release was observed, compared to untreated explants (*p* = 0.999).

#### Cohort 1

**Figure 3:**
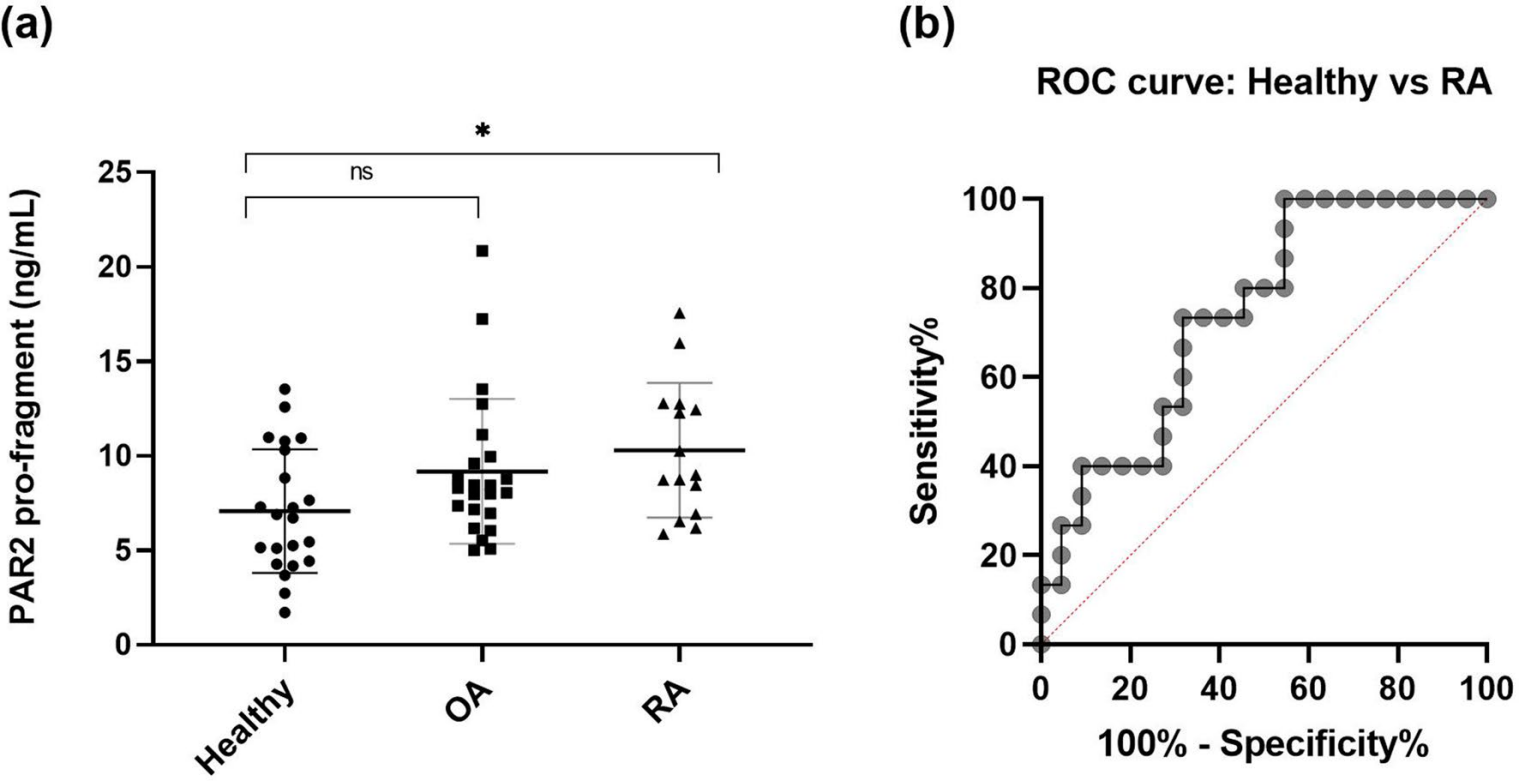
(**a**) Distribution of PRO-PAR2 values across the healthy human donors, OA, and RA patients. Groups were compared using the ANCOVA analysis. Differences between the groups were adjusted to age and gender. Data are presented as mean ± SD. Statistical significance is considered as *P < 0.05, ns = *P* > 0.05. (**b**) Receiver operating characteristics (ROC) curve analysis. This analysis was used to assess the discriminatory performance of PRO-PAR2 levels between controls and RA patients.

To investigate possible clinical relevance the PRO-PAR2 was measured in a discovery cohort (cohort 1), which consisted of 22 healthy controls, 23 OA patients and 15 RA patients (Table 2). Healthy controls and diseased samples were not age and gender-matched, and correction for age and gender was performed with ANCOVA analysis. RA patients showed elevated levels of PRO-PAR2 compared to healthy controls (*p* = 0.043) (Fig. 3a), while no difference was found between RA and OA patients (*p* = 0.852) or OA and healthy subjects (*p* = 0.214). The diagnostic capacity of PRO-PAR2 to identify RA patients from healthy controls showed an AUC of 0.74 (95% CI 0.59–0.90, *p* = 0.013) with a 73.3% sensitivity and 68.2% specificity (Fig. 3b).

**Table 2:** Demographic characteristics for the different cohorts used for the biological validation of the PAR2 immunoassay.

Characteristics	Controls (n = 22)	OA patients (n = 23)	RA patients (n = 15)
Age (range)	37.8 (24–60)	59.3 (41–80)	42.3 (39–47)
Gender, % female	67%	60.9%	53.3%

Data are shown as mean (range).

#### Cohort 2

**Figure 4:**
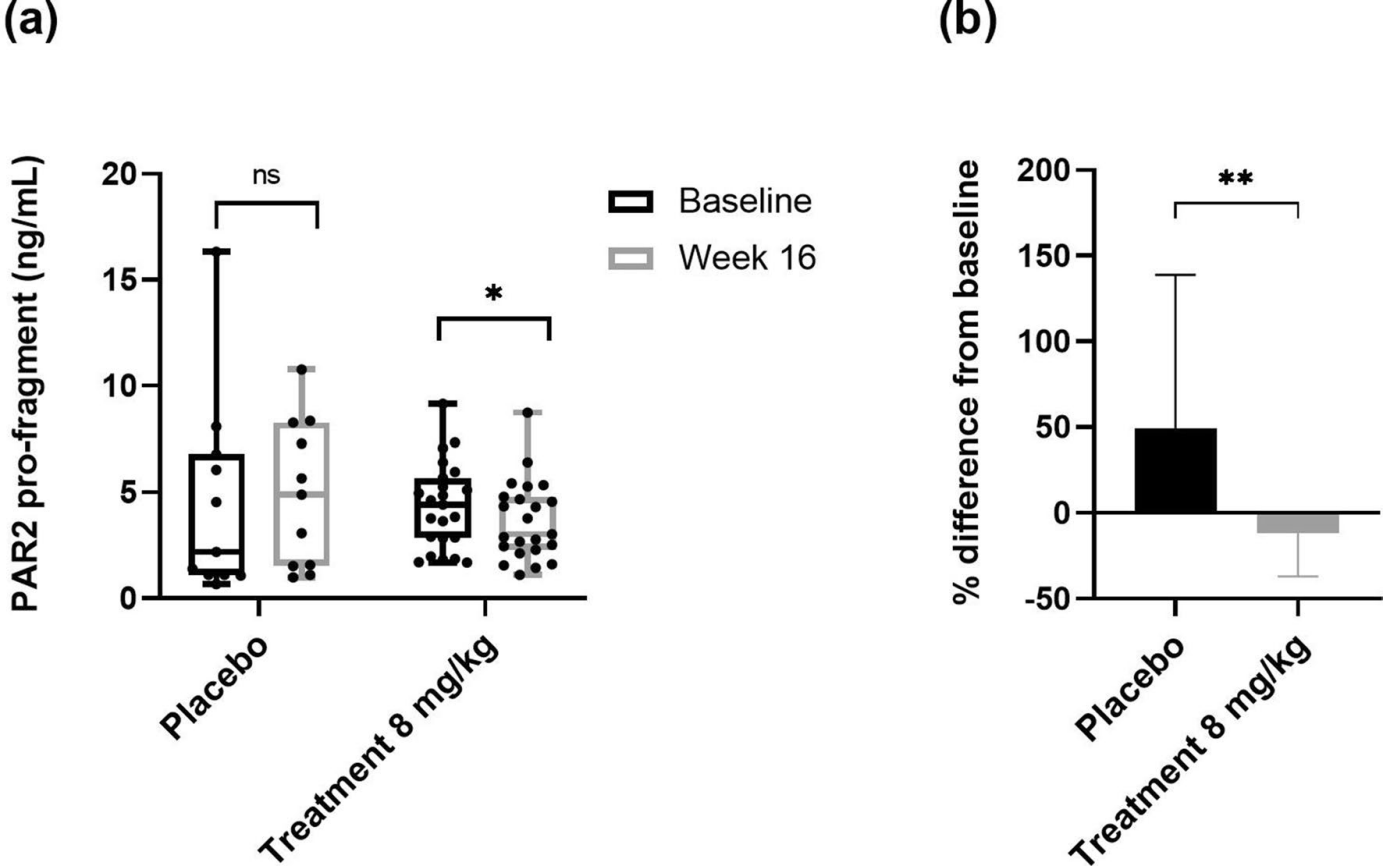
(**a**) PRO-PAR2 levels in placebo and treated patients with tocilizumab at baseline and 16 weeks. PRO-PAR2 levels in the treatment group were compared to the placebo group after 16 weeks. Data are presented as Tukey-Box and whisker plot. Wilcoxon’s paired signed-rank test was used for the comparison. (**b**) Percentage difference from baseline for placebo and treatment group after 16 weeks. Data are presented as mean ± SD. Mann–Whitney test for the percentage change between the placebo and the treatment group was used for comparison. Statistical significance is considered as *P < 0.05, **P < 0.01 and ns = *P* > 0.05.

To further test biological relevance with disease, a subset of the RADIATE study (cohort 2) was measured to test the effect of the anti-IL-6 receptor antibody tocilizumab on the levels of the PRO-PAR2 in circulation. PRO-PAR2 levels were quantified in serum samples from the placebo (n = 11) and 8 mg/kg treatment (n = 23) groups, in patients that had both baseline and 16-week timepoints available. No statistical differences in the demographic characteristics neither between the two groups (placebo and treated) at baseline (Table 3) nor between the subset and the total population were investigated (Supplementary Table S1). In the 8 mg/kg group PRO-PAR2 levels were significantly decreased at week 16 (*p* = 0.049) compared to baseline, while no statistical changes were observed in the placebo group (*p* = 0.275) (Fig. 4a). Furthermore, PRO-PAR2 levels were significantly reduced in 8 mg/kg tocilizumab group compared to placebo at week 16 (− 12% vs + 50% respectively, *p* = 0.007) (Fig. 4b).

**Table 3:** Demographic characteristics for placebo and treated patients of the subset.

Characteristics	Placebo + MTX (n = 11)	Tocilizumab 8 mg/kg (n = 23)	*p* value
Age	53.4 (20–72)	54.9 (20 – 76)	0.779
Gender, % female	81.8%	95.5%	0.252
Ethnicity	72.7% Non-Hispanic 27.3% Hispanic	90.9% Non-Hispanic 9.1% Hispanic	0.304
Race	100% White	95.5% White 4.5% Other	0.999
CRP	4.339 (0.581–24.100)	2.623 (0.113–12.500)	0.299
ESR	51.73 (17–186)	45.64 (8–118)	0.780
HAQ	1.750 (0.375–3.000)	1.517 (0.375–2.750)	0.274
SJC	18.09 (7–39)	18.14 (7–36)	0.858
TJC	28.18 (9–46)	31.73 (8–66)	0.456
VAS PAIN	58.72 (29–79)	63 (21–98)	0.645
DAS	6.649 (4.441–7.702)	6.804 (5.458–8.668)	0.873

Categorical variables are described as percentages, while continuous variables as means (range) and were compared between the two groups (placebo and treated) with Mann–Whitney test. *P* values are shown in the last column. Statistical significance is considered as *P < 0.05, ns = *P* > 0.05. CRP: C-reactive protein, ESR: erythrocyte sedimentation rate, HAQ: health assessment questionnaire, SJC: swollen joint count, TJC: tender joint count, VAS: visual analogue scale, DAS: disease activity score.

## Discussion

Osteoarthritis and rheumatoid arthritis are the most common types of arthritis. Biochemical markers are non-invasive and cost-effective potential tools for measuring disease activity in OA and RA. Although commercial biomarkers are available, there is still an increasing need for sensitive and accurate biomarkers for early diagnosis and treatment monitoring^26^. PAR2 has been found to be associated with the pathology and the progression of arthritic diseases and is considered a therapeutic target not only for disease progression, but also for pain as well^25^.

In this study a novel PAR2 chemiluminescence immunoassay was developed detecting PAR2 pro-fragment (PRO-PAR2) that is released upon PAR2 activation. The main findings of this study were: (1) the novel PAR2 immunoassay was specific towards the neo-epitope and technically robust, (2) PRO-PAR2 was released by matriptase cleavage in vitro and detected by the assay, (3) PRO-PAR2 was released from human synovial membrane explants after treatment with matriptase, (4) PRO-PAR2 levels were increased in RA patients compared to healthy individuals, (5) treatment with tocilizumab led to reduced PRO-PAR2 levels.

Specificity test showed that PAR2 monoclonal antibody was specific for the neo-epitope sequence as there was no reaction neither for the elongated and truncated peptide nor for the full-length PAR2 protein. All technical tests evaluating precision, accuracy, linearity, stability, and interference were acceptable within prespecified limits. We could conclude that this assay is technically robust and reliable for quantitative determination of the PRO-PAR2 in serum. Furthermore, neo-epitope specificity was confirmed with the cleavage experiment where PRO-PAR2 was generated and detected after in vitro cleavage with matriptase. The reduction of PAR2 pro-fragment levels from the 2 to 24-h timepoint shows dynamics in the system and could indicate further cleavage and degradation of the neo-epitope with time. More stability tests for the 36 amino acid pro-fragment need to be performed.

It has been suggested that the PRO-PAR2 may possess signaling properties, although only few studies investigating the role of the released 36-amino-acid fragment have been published. Studies suggest the presence of glycosylation sites, which seem to be involved in cell surface expression and signaling of the receptor^27,28^.

Additionally, the ex vivo experiments showed that PRO-PAR2 was released from treated synovial membrane explants as well. PRO-PAR2 levels were elevated in matriptase-treated explants compared to controls indicating that matriptase can cleave and activate PAR2 in an ex vivo system as well and the PRO-PAR2 can be detected by ELISA. This finding is in agreement with literature, where PAR2 expression is increased in OA fibroblasts^29^.

In our study PRO-PAR2 was significantly increased in RA patients, while a numeric increase was observed in OA patients, which did not reach statistical significance. OA is a heterogeneous disease that may only affect a single or few joints. Thus, the contribution to systemic levels of an analyte may be lower as compared to a systemic inflammatory disease such as RA. In RA it has previously been shown that PAR2 levels are increased in monocytes, macrophages, and fibroblast-like synoviocytes^25^. Increased levels of PRO-PAR2 in serum of RA patients is in line with studies showing increased expression of PAR2 transcript and protein in synovial biopsies and isolated fibroblasts from RA synovium^19^.

PRO-PAR2 levels in serum were measured in a cohort of RA patients treated with methotrexate and tocilizumab and was compared to patients treated with methotrexate only. Tocilizumab blocks inflammatory responses by inhibiting IL-6 receptor activation which we hypothesize may indirectly lead to less PAR2 activation from inflammatory proteases. The link between PAR2 and IL-6 secretion has previously been studied and indicate that PAR2 induces IL-6 secretion from macrophages and T-cells^30,31^. Although PAR2 seems to be an upstream regulator of IL-6 secretion, we could assume that blockage of a pleiotropic cytokine like IL-6, maybe indirectly downregulates PAR2-activating proteinases and thus reduces PRO-PAR2 levels, although this remains speculative. More specifically, IL-6 favors the transition of neutrophiles to monocytes^32^ and the last have been shown to secrete PAR2-activation proteases that initiate inflammatory signaling leading to a sustainable inflammatory loop^33^. Indeed, PRO-PAR2 levels were significantly decreased in patients treated with tocilizumab indicating that PRO-PAR2 may be a potential pharmacodynamic marker to assess pharmacological response to anti-rheumatic and anti-inflammatory drugs. Further clinical studies are needed to validate these findings.

The major limitation of this study is the low number of patients included in the analysis and the exploratory nature of the biological validation. From cohort 2 only a subset of the original study was measured due to limited sample availability. Similarly, cohort 1 consisted of commercial samples and included only few patients. Further investigation and validation in larger cohorts are needed to confirm the findings of this exploratory study. Well-characterized study populations could provide us with more information about the kinetics of the biomarker and association with disease outcomes. Furthermore, the healthy donors’ samples did not come from the same source as the OA and RA patients and some of the patients included in the RA cohort were under treatment. Lastly, regarding the cleavage experiments one limitation is the lack of inhibitor for matriptase deactivation and an inhibitor of PAR2 activation.

## Conclusion

In this study a novel and robust ELISA assay was developed for serological determination of PRO-PAR2 in human serum. The PRO-PAR2 was measured upon matriptase mediated activation of PAR2 both in-vitro and ex vivo. PRO-PAR2 levels were elevated in RA patients compared to healthy controls and were modulated by anti-inflammatory treatment.

## Methods

### Target selection: neo-epitope

**Figure 5:**
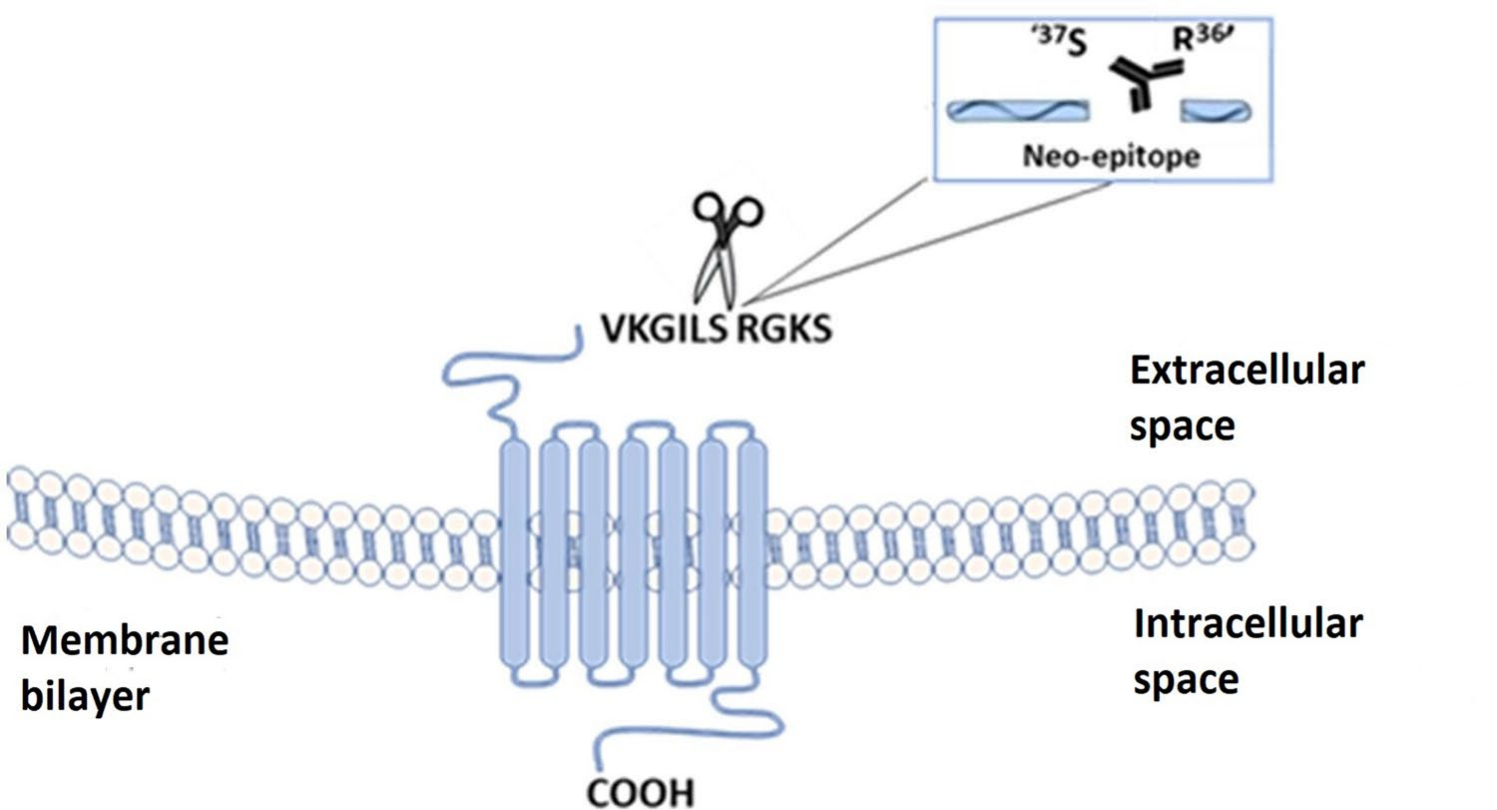
PAR2 receptor activation after cleavage of its N-terminus by serine proteases. The cleavage site between the 36 and 37th amino acid is depicted and the remaining 36-amino-acid fragment (not all 36 amino acids are depicted) is released upon receptor activation.

PRO-PAR2 target sequence is a N-terminal neo-epitope formed after proteolytic cleavage of the full length PAR2 receptor (SKGR36↓37SLIGKV). A sequence of 10 amino acids adjacent to the cleavage site (↓27QGTSRSSKGR36↓) was chosen as the target (Fig. 5). The sequence was analyzed for homology to other human and animal proteins as well as for its uniqueness relative to the entire human proteome by Basic Local Alignment Search Tool (BLAST) using NPS@: Network Protein Sequence Analysis. Synthetic peptides (American Peptide, California, US and GenScript, Piscataway, NJ, US) were used for monoclonal antibody production as well as validation of the immunoassay that was developed. An overview of the peptides used is shown in Table 4.

**Table 4:** Synthetic peptides used for development and validation of the PAR2 immunoassay.

Peptide identifier	Amino acid sequence
Immunogenic peptide	KLH-CGG-QGTNRSSKGR
Biotinylated peptide	Biotin-QGTNRSSKGR
Standard peptide	QGTNRSSKGR
Elongated peptide	QGTNRSSKGRS
Truncated peptide	QGTNRSSKG_

### Generation of monoclonal antibody

For the initiation of the development of this biomarker assay, antibodies were raised against the 10 amino acid sequence ^27^QGTSRSSKGR^35^ next to the cleavage site of PAR2. The method for the generation of monoclonal antibodies has been previously described by Shu et al.^34^. Briefly, 6–7-week-old Balb/C mice were immunized by subcutaneous injection of 200 μL emulsified antigen with 50 μg immunogenic peptide. Immunizations were repeated every 2nd week until stable serum antibody titer levels were obtained. Mice with the highest titer were selected for fusion and boosted intravenously with immunogen 3 days before isolation of the spleen for cell fusion. The mouse spleen cells were following fused with SP2/0 myeloma cells as described by Gefter et al.^35^, to produce hybridoma cells.

After fusion, the clones were plated into 96-well microtiter plates for further growth. Hybridoma cell lines with the highest specificity to the selection peptide were selected for sub-cloning according to the ‘limiting dilution’ method ensuring the growth of single clones. Two clones were used for further growth and for antibody generation. Supernatants from these clones were collected and antibodies purified using protein HiTrap Protein G HP columns according to the manufacturer’s instructions (GE Healthcare Life Sciences, Little Chalfont, UK). The clone with the highest specificity and selectivity was selected for assay development.

### Development and optimization of the PAR2 immunoassay

A competitive chemiluminescence immunoassay (CLIA) was developed with a monoclonal antibody targeting PAR2 pro-fragment (PRO-PAR2). Briefly, the development process included optimization of the concentrations of biotinylated peptide (coater) and detection antibody to achieve the best signal to noise ratio, determination of the optimal buffer concentrations, incubation times and temperatures, generation of the standard curve, determination of the measuring range and technical validation^36^.

In the final assay procedure 96-well streptavidin-coated plates (cat. no. 655995, Greiner Bio-One, Austria) were coated with 100 μL/well of 2 ng/mL biotinylated peptide dissolved in assay buffer (25 mM PBS-BTB, 1% BSA, 0.1% Tween-20, 0.36% Bronidox, 8 g/L NaCl, pH 7.4) and incubated for 30 min at 20 °C in the dark with shaking at 300 rpm. Plates were then washed five times in washing buffer (20 mM TRIS, 50 mM NaCl, pH 7.2). 20 μL of selection peptide, control or sample were added to appropriate wells, followed by 100 μL of horseradish peroxidase (HRP) conjugated monoclonal antibody (final concentration 200 ng/mL). Plates were incubated for 20 h at 4 °C with shaking and followingly washed 5 times in washing buffer. One hundred μL per well of BM Chemiluminescence ELISA Substrate (POD) (cat. no. 11582950001, Roche, Switzerland) solution was added to the plate and incubated for 3 min at 20 °C with shaking. Lastly, the plate was analyzed by a SpectraMax M5 reader (Molecular Devices, CA, USA) with settings: All wavelengths. SoftMax Pro Software was used for data analysis. Sample concentration was determined based on a standard curve generated by serial dilution of the selection peptide and plotted using a 4-parametric mathematical model. Standard concentrations used for the curve were 250, 125, 62.50, 31.25, 15.63, 7.81, 3.91, 1.95, 0.98, 0.49 and 0 ng/mL.

### Technical validation of the PAR2 immunoassay

Different technical tests were performed to assess the quality, robustness, and other parameters of the ELISA assay. The antibody specificity test was performed by assessing reactivity towards the selection peptide, an elongated peptide, and a truncated peptide. Robustness of the assay was evaluated with the intra- and inter-assay variations, with the intra-assay variation to be the mean coefficient of variation (CV%) within the plates and the inter-assay variation the mean CV% among the plates. These variations were calculated after ten independent runs including 7 kit controls, 3 of them were healthy human serum samples and 4 of them were selection peptide spiked in assay buffer. Each run consisted of two replicates of double determinations of the samples. Measurement range was defined as the range between LLMR (lower limit of measurement range) and ULMR (upper limit of measurement range) of the assay and was determined based on the 10 individual standard curves of the intra-inter variation. Apart from the measurement range, the lower limit of detection (LLOD) was determined as the concentration of three standard deviations above the zero standard (buffer).

To assess the linearity of the assay, four healthy human serum samples were used and diluted in assay buffer and the percentage recovery of several dilutions from the samples diluted with assay buffer was calculated. Accuracy was verified by spiking two-fold dilutions of the 36-amino-acid full length pro-peptide in three healthy human serum samples. The recovery was determined as the percentage recovery of the expected concentration, after combining the concentration of the analyte present in serum sample and the peptide concentration. The analyte stability was determined for three healthy human serum samples which were incubated at either 4 °C or 20 °C for 2, 4, 24 and 72 h, respectively. One more test was performed to assess the stability of the samples by subjecting them to four freeze and thaw cycles and calculating the percentage recovery using the zero freeze/thaw cycle as reference samples. Finally, interference was measured in healthy human serum spiked with two different concentration of biotin (low = 5 ng/mL, high = 100 ng/mL), hemoglobin (low = 2.5 mg/mL, high = 5.0 mg/mL), or lipids (low = 1.5 mg/mL, high = 5.0 mg/mL) and calculated as the percentage recovery of the analyte in non-spiked serum.

### In vitro cleavage of PAR2

In vitro cleavage with recombinant human PAR2 as the substrate (cat.no. H00002150-P01, Novus Biologicals, Centennial, Colorado, USA) and matriptase as the enzyme (cat.no. 3946-SEB-010, R&D systems, Minneapolis, Minnesota, USA,) was performed. Cleavage buffer for the reaction contained 50 mM Tris-HCL, 50 mM NaCL and 0.01% Tween 20. Cleavage buffer only, PAR2 recombinant protein in cleavage buffer, and matriptase in cleavage buffer without PAR2 protein were used as controls. All ingredients were mixed thoroughly and incubated for 2 h and 24 h at 37 °C. Cleavage products were stored at − 20 °C until further analysis.

### Human synovial explants (HEX) model

Explants were isolated from synovial membranes of OA patients who underwent total knee replacement. The use and the methodology of HEX experiments have been previously described^37^. Briefly, explants were cultured for 21 days with media being changed every 2–3 days and the following treatments were added accordingly: (1) medium without any treatment (w/o), (2) OSM [10 ng/mL] combined with TNF-α [20 ng/mL] (O + T), (3) recombinant human matriptase [30 nM] (matriptase), 4) O + T combined with matriptase (O + T + matriptase). The culture media were harvested every 2–3 days and stored until biochemical analysis. Metabolic activity was evaluated by the AlamarBlue assay (Thermo Fisher Scientific, Waltham, MA, USA) on day 0, 14, and day 21 when the experiment was terminated.

### Biological validation

#### Cohort 1

Serum samples from individuals with OA and RA were acquired from Discovery Life Science (AL, USA). Serum from healthy donors were used as controls and were obtained from BioIVT (West Sussex, UK). All samples were processed immediately after collection according to standard operating procedures and stored at − 80 °C for long-term storage. Samples were measured un-diluted in duplicates and CVs were below 15%.

#### Cohort 2

A subset of serum samples from a phase III RA clinical trial was measured. The RADIATE study has been previously described by Karsdal et al.^38^ (clinicalTrials.gov identifier: NCT00106522). This study is a randomized, double-blind, placebo-controlled, parallel-group phase III trial with RA patients. Patients were randomly assigned to either tocilizumab (4 or 8 mg/kg) or placebo intravenously every 4 weeks, along with concomitant stable methotrexate (10 to 25 mg weekly) in all treatment groups. Clinical assessment and blood samples were obtained at baseline and after 16 weeks of follow-up. Samples were processed immediately and stored at − 80 °C until analysis. In the current study only samples from the placebo and the 8 mg/kg treatment groups were used. Furthermore, only patients with remaining baseline and follow up sample were included. Samples were measured in duplicates with CV below 15%. Due to unavailability of material a subset of patients was measured and any statistical differences in the demographic characteristics between the two groups (total and subset) at baseline were investigated. Differences in the demographic characteristics between placebo and treated patients were investigated as well.

### Statistical analysis

Means, standard deviations, medians, and ranges were used to describe continuous variables, while counts/frequencies and percentages were used to describe categorical variables. Statistical differences between categorical variables were assessed using Chi-square test. For the cleavage experiment, one-way ANOVA with Dunnett’s multiple comparison test was performed to assess the difference in the mean levels of the PRO-PAR2 in the different experimental groups setting as control the protein-only (PAR2) group. Area under the curve (AUC) from treated synovial explants was compared to the untreated group using Kruskal–Wallis test with Dunn’s post-hoc test for multiple comparisons. For continuous variables, analysis of covariance (ANCOVA) was performed to examine the difference in mean levels of PRO-PAR2 among healthy controls, OA, and RA patients adjusting for age and gender. Receiver operating characteristics (ROC) analysis was performed to assess the diagnostic accuracy of the PRO-PAR2. Wilcoxon’s paired signed-rank test was used to determine if there is a difference in mean levels of PRO-PAR2 between baseline and week 16 for the 2 different groups (placebo and treated). Mann–Whitney test was performed to assess the difference in the percentage change from baseline between placebo and treated patients. For all statistical analysis performed, statistical significance was set to 0.05. For the analysis of the data GraphPad Prism, version 8.3.0 (GraphPad Software, Inc., CA, USA) and R version 4.0.3 were used.

### Ethical statement

The production of monoclonal antibodies performed in mice was approved by the Danish Animal Ethics Council under approval number 2013–15-2934–00,956. All animal experiments were carried according to relevant guidelines and regulations and all protocols were performed according to ARRIVE guidelines. The use of human tissues for the ex vivo experiments was approved by the Danish Scientific Ethics Committee for the Capital Region of Denmark (Den Videnskabsetiske Komité for Region Hovedstaden) under permit number H-D-2007–0084. All experimental protocols were performed according to relevant guidelines and regulations and informed consent was obtained from all participants. Healthy, OA and RA serum samples used for biological validation were commercially available and were ethically obtained, following all applicable HHS/OHRP, ISBER, and NCI/BBRB regulations, guidelines, and best practices. For the collection of samples informed consent was obtained from all the participants. The treated RA serum samples were subset of a clinical study (RADIATE study). The study was conducted throughout North America and Western Europe and was approved by institutional review boards, ethics committees and regulatory authorities. Informed consent was obtained from each patient in compliance with the Helsinki Declaration, and the study was registered at ClinicalTrials.gov (NCT00106522).

## Supplementary Information

Supplementary Information.

## Data Availability

The data generated during this study are available from the corresponding author upon reasonable request.
