# Supplementary material for: The activation fragment of PAR2 is elevated in serum from patients with rheumatoid arthritis and reduced in response to anti-IL6R treatment
Source: Sci Rep. 2021 Dec 20;11:24285. doi: 10.1038/s41598-021-03346-0 (PMC8688421; doi:10.1038/s41598-021-03346-0)
Supplement: Supplementary file 1 — Supplementary Information. [file 41598_2021_3346_MOESM1_ESM.docx]

Supplementary material

***Supplementary Table S1: Demographic characteristics for subset and total population for each group (placebo and treated).***

| *Characteristics* | *Placebo + MTX*  *subset (n=11)* | *Placebo + MTX*  *total (n=159)* | *p-value* | *Tocilizumab 8mg/kg*  *subset (n=23)* | *Tocilizumab 8mg/kg*  *total (n=174)* | *p-value* |
| --- | --- | --- | --- | --- | --- | --- |
| *Age* | 53.4  (20 – 72) | 53.6  (20 – 83) | 0.7643 | 54.9  (20 – 76) | 53.9  (21 – 82) | 0.881 |
| *Gender,*  *% female* | 81.8% | 78.8 % | 0.999 | 95.5% | 83.4 | 0.210 |
| *Ethnicity* | 72.7% Non-Hispanic  27.3% Hispanic | 88.8% Non-Hispanic  27.3% Hispanic | 0.124 | 90.9% Non-Hispanic  9.1% Hispanic | 86.8% Non-Hispanic  13.2% Hispanic | 0.746 |
| *Race* | 100% White | 94.4% White  5.6% Other | 0.495 | 95.5% White  4.5% Other | 89.1% White  10.9% Other | 0.706 |
| *CRP* | 4.339  (0.581 – 24.100) | 2.320  (0.02 – 24) | 0.8170 | 2.623  (0.113 – 12.500) | 3.146  (0.02 – 18.50) | 0.248 |
| *ESR* | 51.73  (17 – 186) | 54.6  (9 – 186) | 0.3556 | 45.64  (8 – 118) | 48.94  (5 – 133) | 0.345 |
| *HAQ* | 1.750  (0.375 – 3.000) | 1.704  (0 – 3) | 0.8228 | 1.517  (0.375 – 2.750) | 1.738  (0.375 – 2.875) | 0.181 |
| *SJC* | 18.09  (7 – 39) | 18.86  (4 – 50) | 0.9492 | 18.14  (7 – 36) | 18.74  (6 – 56) | 0.832 |
| *TJC* | 28.18  (9 – 46) | 30.47  (5 – 68) | 0.7714 | 31.73  (8 – 66) | 31.53  (8 – 67) | 0.734 |
| *VAS PAIN* | 58.72  (29 – 79) | 64.22  (0 – 100) | 0.373 | 63  (21 – 98) | 64.64  (10 – 100) | 0.567 |
| *DAS* | 6.649  (4.441 – 7.702) | 6.8  (3.617 – 8.943) | 0.6871 | 6.804  (5.458 – 8.668) | 6.780  (4.558 – 8.668) | 0.974 |

*Categorical variables are described as percentages, while continuous variables as means (range) and were compared between subset and total population for each group (placebo and treated) with Mann-Whitney test. P-values are shown separately for the placebo and the treated (tocilizumab 8mg/kg) group. Statistical significance is considered as *=P<0.05, ns=P>0.05. CRP: C-reactive protein, ESR: erythrocyte sedimentation rate, HAQ: health assessment questionnaire, SJC: swollen joint count, TJC: tender joint count, VAS: visual analogue scale, DAS: disease activity score.*
